# Breastfeeding after Returning to Work: A Systematic Review and Meta-Analysis

**DOI:** 10.3390/ijerph18168631

**Published:** 2021-08-15

**Authors:** Frédéric Dutheil, Grégory Méchin, Philippe Vorilhon, Amanda C. Benson, Anne Bottet, Maëlys Clinchamps, Chloé Barasinski, Valentin Navel

**Affiliations:** 1 CNRS, LaPSCo, Physiological and Psychosocial Stress, University Hospital of Clermont-Ferrand, CHU Clermont-Ferrand, Occupational and Environmental Medicine, Université Clermont Auvergne, WittyFit, F-63000 Clermont-Ferrand, France; maelysclinchamps@gmail.com; 2 Department of General Practice, UFR Medicine, 28 Place Henri-Dunant, Université Clermont Auvergne, F-63000 Clermont-Ferrand, France; gregory.mechin@gmail.com; 3 Department of General Practice, UFR Medicine, Research Unit ACCePPT Self-Medication, Multi-Professional Support for Patients, Université Clermont Auvergne, 28 Place Henri-Dunant, F-63000 Clermont-Ferrand, France; pvorilhon2@wanadoo.fr (P.V.); anne.bottet@uca.fr (A.B.); 4 Swinburne University of Technology, Health and Biostatistics, Hawthorn, Victoria, VIC 3122, Australia; abenson@swin.edu.au; 5 CNRS, SIGMA Clermont, Institut Pascal, University Hospital of Clermont-Ferrand, CHU Clermont-Ferrand, Université Clermont Auvergne Perinatality, F-63000 Clermont-Ferrand, France; cbarasinski@chu-clermontferrand.fr; 6 CNRS, INSERM, GReD, CHU Clermont-Ferrand, University Hospital of Clermont-Ferrand, Ophthalmology, Université Clermont Auvergne, F-63000 Clermont-Ferrand, France; valentin.navel@hotmail.fr

**Keywords:** lactation, occupation, public health, pregnancy, well-being

## Abstract

Background: The benefits of breastfeeding are widely known; however, continuation after returning to work (RTW) is not. We aimed to conduct a systematic review and meta-analysis to assess the prevalence of breastfeeding after RTW. The secondary objectives were to compare the economic statuses between continents. Method: PubMed, Cochrane Library, Base, and Embase were searched until 1 September 2020, and two independent reviewers selected the studies and collated the data. To be included, articles needed to describe our primary outcome, i.e., prevalence of breastfeeding after RTW. Results: We included 14 studies, analyzing 42,820 women. The overall prevalence of breastfeeding after RTW was 25% (95% CI, 21% to 29%), with an important heterogeneity (I^2^ = 98.6%)—prevalence ranging from 2% to 61%. Stratification by continents and by GDP per capita also showed huge heterogeneity. The Middle East had the weakest total prevalence with 10% (6% to 14%), and Oceania the strongest with 35% (21% to 50%). Despite the prevalence of breastfeeding in general increasing with GDP per capita (<US$5000: 19%, US$5000–30,000: 22%; US$30,000 to 50,000: 25%, >US$50,000 42%), the prevalence of non-exclusive breastfeeding follows more of a U-curve with the lowest and highest GDP per capita having the highest percentages of breastfeeding (<US$5000: 47% and >US$50,000: 50%, versus <28% for all other categories). Conclusion: Breastfeeding after RTW is widely heterogeneous across the world. Despite economic status playing a role in breastfeeding after RTW, cultural aspects seem influential. The lack of data regarding breastfeeding after RTW in most countries demonstrates the strong need of data to inform effective preventive strategies.

## 1. Introduction

Breastfeeding provides multiple health advantages for the child (infections, malocclusion, and intelligence) and their mother (breast cancer) [1,2,3,4], with economic and social benefits as well (cost savings for parents, employers, and society, as well as the parent–child relationship) [3,5,6,7]. Hence, the World Health Organization (WHO) recommends “exclusive breastfeeding for the first 6 months of life and introduction of nutritionally-adequate and safe complementary (solid) foods at 6 months together with continued breastfeeding up to 2 years of age or beyond” [8]. During this breastfeeding transition time, returning to work (RTW) is common for mothers who have to manage work and breastfeeding. RTW represents one of the main reasons for stopping breastfeeding [9,10,11,12]. Combining breastfeeding and work may be hard for mothers depending on their working conditions [13], sociocultural heritage and gender role ideology [14], public health policies [15], and economy and lobby groups [16]. For example, in a Taiwanese study, 67% of working mothers initiated breastfeeding, but only 10% continued after RTW [17]. Both the culture of work and breastfeeding differ between countries; for example, breastfeeding initiation may vary from 47% (Ireland) to 99% (Norway) [18] within developed European countries. In addition to breastfeeding initiation, the type of breastfeeding (exclusive or non-exclusive) may also be at the interplay between the work environment and sociocultural/economic aspects [19]. However, no studies have summarized the differences in breastfeeding after RTW or have compared countries. Conversely, women from low-income countries have difficulty combining work and breastfeeding [20], and therefore might be at risk of ceasing breastfeeding when returning to work. Considering the importance of breastfeeding, an evidence-based study is needed to summarize the existing literature for building efficient promotion and support for breastfeeding in the workplace. 

Therefore, we aimed to conduct a systematic review and meta-analysis to evaluate the prevalence of breastfeeding after RTW (primary aim). The secondary objectives were to evaluate the differences between continents or their level of development, as well as putative influencing variables such as sociodemographics [21,22,23], breastfeeding support at work [24,25,26,27], or workplace policy [28,29,30]. Additionally, we evaluated the influence of the previous factors on the type of breastfeeding (exclusive or not).

## 2. Methods

### 2.1. Literature Search

**Figure 1:**
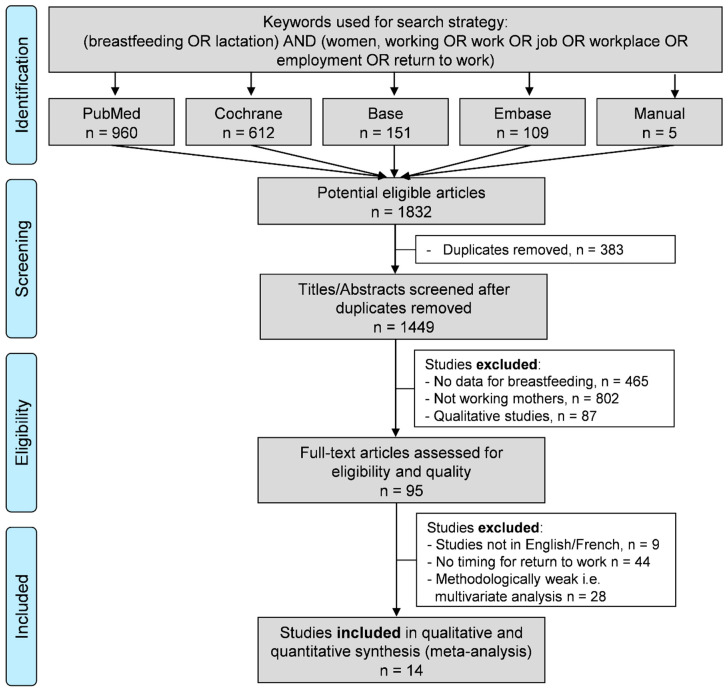
Search strategy.

We reviewed all studies involving breastfeeding after returning to work. Specifically, the inclusion criteria for the search strategy were the prevalence of breastfeeding and/or exclusive breastfeeding after RTW, using the following keywords: Breastfeeding AND work (see detailed search strategy in Appendix A.1). The following databases were searched on 1 September 2020: PubMed, Cochrane Library, Embase. and Base. The search was not limited to specific years. To be included, articles needed to describe our primary outcome variable, which was the prevalence of breastfeeding after RTW, i.e., women had to have returned to work and studies had to have reported the timing of RTW. Specifically, we excluded studies when mothers did not work, or did not describe breastfeeding and its timing related to RTW. Studies that were not written in English or French were also excluded, as well as qualitative studies. In addition, reference lists of all publications meeting the inclusion criteria were manually searched to identify any further studies not found through electronic searching. The PRISMA flow diagram of the search strategy is presented in Figure 1. Two authors (G. Méchin and M. Clinchamps) conducted all of the literature searches, as well as collated and independently reviewed the abstracts. Based on the selection criteria, they decided the suitability of the articles for inclusion. A third author (F. Dutheil) was asked to review the articles where consensus on suitability was debated. Then, all authors reviewed the eligible articles. We followed the PRISMA guidelines (Appendix A.2) [31].

### 2.2. Data Collection

The data collected included the authors’ name, publication year, study design, duration of studies, aims, outcomes of the included articles, sample size, mean age, occupation, countries and continents, and their economic status (gross domestic product (GDP) per capita), month of RTW, breastfeeding practices (global, exclusive, or non-exclusive), and characteristics of the individuals (such as education, birth delivery, and smoking) (Table 1).

### 2.3. Quality of Assessment

An assessment of the methodological quality was performed using the Newcastle–Ottawa Scale (NOS) for cohort studies [32] and modified NOS for cross-sectional studies [33]. The items assessed were selection bias (four items), comparability bias (one item), and outcome bias (three items for cohort and two for cross-sectional studies). Each item was assigned a judgment of “Yes” (1 point), “No” (0 point), or “Can’t say” (0 point). Thus, the maximum score was 8 points for cohort studies and 7 points for cross-sectional studies (Appendix A.3 and Appendix A.4). Disagreements between reviewers (G. Méchin and M. Clinchamps) were addressed by obtaining a consensus with a third author (F. Dutheil).

### 2.4. Statistical Considerations

Statistical analysis was conducted using Stata software (version 15, StataCorp, College Station, TX, USA) [34,35,36,37,38,39,40,41]. The characteristics of breastfeeding, work, the individuals, or other variables were summarized for each study sample and reported as the mean ± standard deviation (SD) and number (%) for continuous and categorical variables, respectively. Random effects meta-analyses (DerSimonian and Laird approach) on the prevalence of breastfeeding after RTW were conducted when the data could be pooled [42]. *p*-Values less than 0.05 were considered statistically significant. We stratified these meta-analyses by continents and by economic status of the countries (GDP per capita). All of these meta-analyses were computed for global, exclusive, and non-exclusive breastfeeding. Heterogeneity in the study results was evaluated by examining forest plots and confidence intervals (CIs) and by using formal tests for homogeneity based on the I^2^ statistic, which is the most common metric for measuring the magnitude of between-study heterogeneity and is easily interpretable. I^2^ values range between 0% and 100% and are typically considered low for <25%, modest for 25%–50%, and high for >50% [42]. For example, significant heterogeneity may be due to the variability between the characteristics of the studies, such as the type of breastfeeding (exclusive or not), occupational settings, or the characteristics of the countries or individuals. For thoroughness, funnel plots of these meta-analyses were used to search for potential publication biases. In order to verify the strength of the results, further meta-analyses were then conducted, excluding studies that were not evenly distributed around the base of the funnel [43]. When possible (sufficient sample size), meta-regressions were proposed to study the relationship between the prevalence of breastfeeding after RTW and putative variables such as continent, economic status of countries (GDP), or characteristics of the individuals (age, education, etc.). The results are expressed as regression coefficients and 95% CIs.

## 3. Results

An initial search produced a possible 1832 articles (Figure 1). Removal of duplicates (*n* = 383) and applying the selection criteria reduced these articles reporting the prevalence of breastfeeding after RTW to 14 studies (Figure 1) [44,45,46,47,48,49,50,51,52,53,54,55,56,57]. All of the identified articles were written in English (Table 1).

### 3.1. Quality of the Articles

The quality assessment of the 14 included studies, as outlined by the NOS, varied from 57.1% [57] to 100% [44], with a mean score of 81.8 ± 7.9%. The most frequent biases were the assessment of outcomes (self-reported) for cohort studies and the selection, especially considering the limited sample size in some studies. There was also a lack of follow-up in the cohort studies. Detailed characteristics of methodological quality assessment of each included study are available in Appendix A.3 and Appendix A.4. All studies mentioned ethical approval. 

### 3.2. Population

**Sample size**: Population sizes ranged from 84 [45] to 20,172 [49]. In total, 42,820 women were included in this meta-analysis.

**Age:** All studies reported age. Seven studies reported mean age [46,49,50,51,52,54,56], ranging from 26.9 [54] to 33 years [56], and seven studies reported a cut-off for age [44,45,47,48,53,55,57] from <25 to >30 years old. 

**Gender:** All studies included only women (42,820 in total).

**Type of occupation:** Nine studies included all working mothers with no job specification [46,47,48,49,50,52,53,54,55]. Two studies included employed workers in formal and informal sectors [51,57]. One study included mothers who were professional/semi-professional, manual, or business workers [44]. One study included mothers in paid employment [56]. One study included government and semi-government employees, private company employees, and self-employed or family business owners [45].

**Country of breastfeeding:** Two studies were conducted in Europe (France [47] and the United Kingdom [51]), two studies in the Middle East (Israel [46] and Egypt [44]), three in the United States of America [52,53,54], four in Asia (Thailand [45,57], India [48], and Taiwan [49]), and three in Oceania (Australia [50,55,56]).

**Gross domestic product per capita:** We retrieved the GDP per capita by country and year of the included studies using data from the World Bank database [58].

**Other characteristics:** Characteristics such as education [44,47,48,49,50,52,53,54,56,57], mode of delivery [44,45,48,49,50,52,53,55], and smoking status [47,50,52,53,55] were inconsistently reported, precluding further analyses (Table 1).

### 3.3. Inclusion and Exclusion Criteria within the Included Articles

Working mothers were the shared inclusion criterion for the 14 studies [44,45,46,47,48,49,50,51,52,53,54,55,56,57]. Six studies included working mothers who had regular work over the 12 months prior to birth [44,48,52,53,56,57]. Two studies specified that charitable work was excluded [45,49]. Two studies only included single mothers [51,53], with one restricting inclusion to British/Irish white natural mothers [51]. Two studies only included infants free of any serious health conditions [50,55]. Three studies only included mothers who initiated breastfeeding [47,52,54] prior to RTW. The exclusion criteria were a severe illness, either in the mother or the baby [45,48,49], mothers of twins [44], and mothers who never initiated breastfeeding [56].

### 3.4. Outcome and Aim of the Studies

The primary outcome of the included articles was the prevalence of breastfeeding after RTW for six studies [44,46,49,50,53,56], and the duration of breastfeeding for two studies [47,51]. The other studies aimed to assess the factors related to breastfeeding at work [45,48,52,54,55,57].

### 3.5. Study Designs

Seven studies had a cross-sectional prevalence survey design, analyzing breastfeeding amongst working mothers [44,45,46,48,50,56,57]. Seven studies had a cohort follow-up design [47,49,51,52,53,54,55], analyzing the prevalence of breastfeeding after RTW over time [47,49,51,53,55] or from survey data [52,54].

### 3.6. Breastfeeding and Return to Work

**Method of assessment:** Breastfeeding after RTW was measured via a questionnaire [47,50], semi-structured interview questions [44,45,48,49,57], telephone [55,56], or at home [46,51,53]. Two studies retrieved breastfeeding prevalence using survey data at follow-up [52,54].

**Type of breastfeeding:** Seven studies investigated both exclusive and non-exclusive breastfeeding [47,50,53,54,55,56,57], only two reported exclusive breastfeeding [44,48], and only five reported non-exclusive breastfeeding [45,46,49,51,52].

**Return to work:** RTW after birth ranged from <1 month [49] to 12 months [49,50,55]. The heterogeneous time of RTW precluded stratification of breastfeeding by month of RTW (Table 1).

### 3.7. Meta-Analysis on the Prevalence of Breastfeeding after Returning to Work

**Figure 2:**
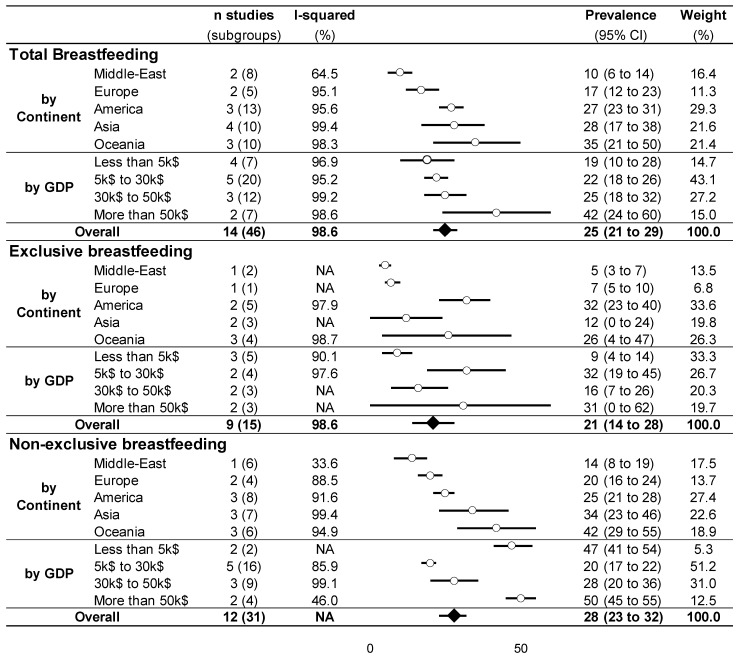
Meta-analysis of the prevalence of breastfeeding.

Our meta-analysis demonstrated an overall prevalence of breastfeeding after RTW of 25% (95% CI, 21% to 29%), with an important heterogeneity (I^2^ = 98.6%)—the prevalence of breastfeeding after RTW ranging from 2% [48] to 61% [45]. Stratification by continents (Appendix A.5) and by GDP per capita (Appendix A.6) also showed large heterogeneity. Middle Eastern countries had the weakest total prevalence with 10% (6% to 14%), and Oceania (Australia) the strongest with 35% (21% to 50%). The prevalence of breastfeeding was 19% (10% to 28%) for GDP under US$5000 per capita, 22% (18% to 26%) between US$5000 and US$30,000, 25% (18% to 32%) between US$30,000 and US$50,000, and 42% (24% to 60%) for GDP higher than US$50,000 (Figure 2).

Similarly, the meta-analysis on exclusive and non-exclusive breastfeeding showed high heterogeneity (I^2^ > 90%), with a mean overall prevalence of breastfeeding after RTW of 21% (14% to 28%) and 28% (23% to 32%), respectively. Stratification by continents demonstrated similar results, with Middle Eastern countries having the weakest prevalence (5% (3% to 7%) and 14% (8% to 19%), respectively) and Oceania countries the strongest (26% (4% to 47%) and 42% (29% to 55%), respectively). Stratification by GDP did not show an increase in exclusive or non-exclusive breastfeeding with the economic status of countries. For example, for non-exclusive breastfeeding, the highest prevalence of breastfeeding was for the lowest and highest GDP (47% (41% to 54%) for GDP under US$5000 per capita and 50% (45% to 55%) for GDP higher than US$50,000, whereas the prevalence was 20% (17% to 22%) between US$5000 and US$30,000 and 28% (20% to 36%) between US$30,000 and US$50,000) (Figure 2). 

### 3.8. Sensitivity Analysis and Other Meta-Regressions

**Figure 3:**
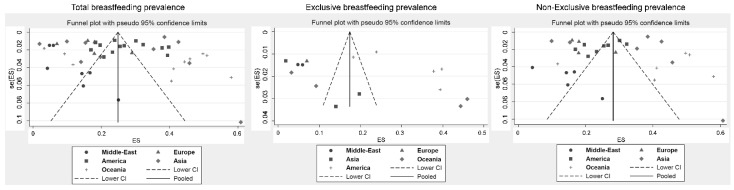
Meta-funnels.

**Figure 4:**
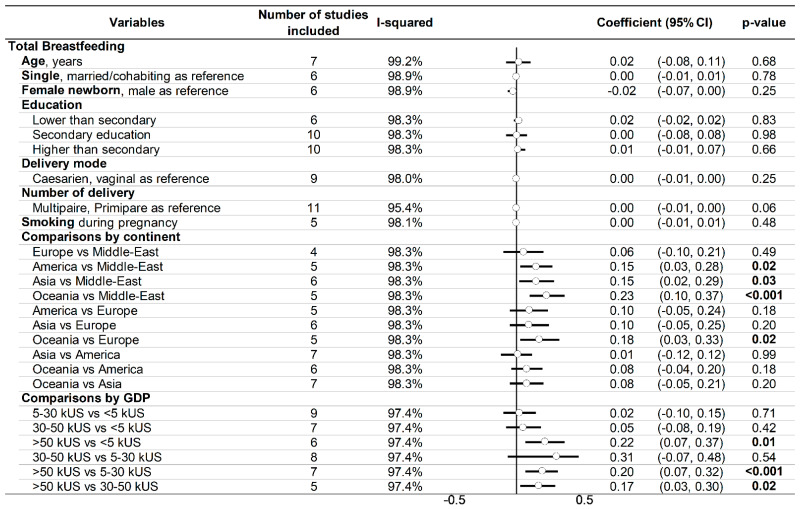
Meta-regression.

Funnel plots of these meta-analyses demonstrated a wide heterogeneity (Figure 3), precluding any sensitivity analyses, with most studies being outside of the meta-funnels. For overall and non-exclusive breastfeeding, meta-regressions by continent demonstrated a lower prevalence of breastfeeding in the Middle East compared to Asia (coefficient = 0.15, 95% CI = 0.02 to 0.29 and 0.19, 0.05 to 0.33, respectively) and Oceania (0.28, 0.13 to 0.42 and 0.23, 0.10 to 0.37, respectively) and was also higher in Oceania vs. Europe (0.18, 0.03 to 0.33 and 0.22, 0.07 to 0.37, respectively). The prevalence of overall breastfeeding was also lower in the Middle East compared to the United States of America (0.15, 0.03 to 0.28), and the prevalence of non-exclusive breastfeeding was also higher in Oceania vs. the United States of America (0.18, 0.05 to 0.30). The meta-regressions did not show any exclusive significant association by continent. For overall and non-exclusive breastfeeding, the meta-regressions demonstrated a higher prevalence of breastfeeding for the countries with the highest GDP (>US$50,000) than those with GDP between US$5000 and US$30,000 (0.20, 0.07 to 0.32 and 0.31, 0.2 to 0.41, respectively), between US$30,000 and US$50,000 (0.17, 0.03 to 0.3 and 0.22, 0.11 to 0.33, respectively), and <US$5000 (0.22, 0.07 to 0.37, but only for overall breastfeeding). For non-exclusive breastfeeding, those countries with the lowest GDP (<US$5000) also had a higher prevalence of breastfeeding than countries with GDP between US$5000 and US$30,000 (0.31, 0.15 to 0.48) and between US$30,000 and US$50,000 (0.23, 0.06 to 0.40). The meta-regressions did not demonstrate any influence of individual characteristics (age, education, etc.) (Figure 4).

## 4. Discussion

The main finding was that the prevalence of breastfeeding after RTW is widely heterogeneous across the world. Despite the review demonstrating that economic status may play a role in breastfeeding after RTW, cultural aspects seem an important determinant. We did not find an effect of putative influencing variables.

### 4.1. Breastfeeding around the World

This study is the first meta-analysis analyzing breastfeeding prevalence after RTW. As stated by the WHO, breastfeeding confers various benefits for infants and mothers [59]. However, RTW is one of the major causes (20%) of women stopping breastfeeding, along with fatigue (22%) and insufficient milk supply (21%) [60]. The intention to breastfeed is negatively associated with RTW [61]. The dominant trends of our meta-analysis were heterogeneity and lack of data. We demonstrated a huge heterogeneity in breastfeeding after RTW between and within continents. Even within industrialized European countries, comparisons between countries were available mainly for breastfeeding initiation and duration with a large heterogeneity. For example, France and the U.K. are among the countries with the lowest initiation (62% and 70%, respectively [62]) and prevalence at 12 months [3], whereas Scandinavian countries have the highest initiation (99% for Denmark and Norway [62]) and long-term prevalence. The results from our meta-analysis seemed to show a higher rate of breastfeeding after RTW in Asia than in Europe, in line with the literature (almost 100% of breastfeeding initiation in Myanmar, for example [63]). The United States of America seems to have a similar breastfeeding rate after RTW to Asia. Oceania, represented by Australia, has high rates of breastfeeding after RTW, in line with their goal by 2022 of 40% exclusive breastfeeding until newborns are six months old [64]. Middle Eastern countries have the lowest prevalence of breastfeeding after RTW, in line with their low breastfeeding initiation rate of only one-third of newborns, falling to 20% at six months, without considering returning to work [65]. Even if breastfeeding in general has been widely studied, we demonstrated that data are scarce regarding breastfeeding after RTW in most countries, particularly in some continents such as Africa, where no data are available, demonstrating the urgent need for data from these countries to inform effective preventive strategies. It is known that the cultural aspect is very important for breastfeeding uptake [19]. Mothers’ mothers have a strong positive attitude toward breastfeeding when they are positively reinforced or supported [66]. Notably, highly educated Chinese grandmothers were associated with decreased exclusive breastfeeding in their daughters [67]. This fact could be linked with gender role ideology that varies markedly across countries [68]. Moreover, social and cultural attitudes have an impact on the representation of breastfeeding within and between different countries/continents. A meta-analysis found that community-based interventions, including group counselling or education and social mobilization, with or without mass media, are effective at increasing timely breastfeeding initiation by 86% and exclusive breastfeeding by 20% [19].

### 4.2. Cultural Aspect in Breastfeeding

Interestingly, whatever their economic status, some countries have strong breastfeeding policies, especially after RTW [69]. Australia developed breastfeeding reference groups [70], maternity leave policies [70], and support clinics [71] with home visiting programs [72]. Maternity leave also positively impacts breastfeeding duration [10,12,73,74]. A recent review showed a positive relationship between maternity leave length and breastfeeding duration [75]. Australia, along with Austria and New Zealand, also have high female part-time employment [3], more compatible with breastfeeding after RTW [76]. Moreover, a recent study in Australia highlighted that women’s emotional well-being is related to breastfeeding [77], which may in turn improve well-being at work. In comparison, some developing countries are also culturally prone to breastfeeding, such as Thailand or Myanmar, who regularly promote breastfeeding support assistance after RTW [78,79]. Similarly, 50% of women continue to breastfeed until their child reaches two years of age in Laos and Indonesia, and almost 65% in Myanmar [63]. Our meta-analysis also suggested that exclusive breastfeeding is lower after RTW than non-exclusive breastfeeding. Not surprisingly, combining breastfeeding and work necessitates adaptation—such as the introduction of infant formula, which is very common in countries such as Indonesia [15]. The frequency of infant formula use in Asia may also explain the U-shape of the prevalence curve of non-exclusive breastfeeding (lowest and highest GDP per capita having the highest percentages of breastfeeding). Some working conditions, such as shift work, add difficulties for mothers to exclusively breastfeed their infant [13]. Breastfeeding can also be at the interplay between public health policies, the economy, and lobby groups. In the USA, the Infant Formula Council historically lobbied against the public health promotion of breastfeeding [16], even discouraging a pro-breastfeeding campaign in 2007 [80]. In 2009, only 23/50 states in the USA encouraged workplace breastfeeding by adopting laws, and no state required employers to provide breastfeeding pumping equipment to their employees [81]. In 2011, the USA ranked last out of 36 countries for its breastfeeding policy [16]. Eager to improve workplace lactation, the USA launched ambitious programs [82] that included reasonable break times and adequate space for nursing mothers to express milk [83]. More broadly, the Lancet breastfeeding series highlighted that the promotion of breastfeeding is a collective societal responsibility and not the sole responsibility of an individual woman [19]. One of the six call to action points was to foster positive societal attitudes toward breastfeeding, such as adequate maternity leave and the opportunity to breastfeed or express milk in the workplace [19].

### 4.3. Other Factors Influencing Breastfeeding after Returning to Work

We did not find other factors that influenced breastfeeding prevalence after RTW. Obviously, the literature showed that early RTW negatively affected breastfeeding initiation [84] and duration [73,85], as well as full-time work [10,30]. On the contrary, part-time work has been found to have a positive impact on breastfeeding duration [86,87]. Flexibility in working schedules may be associated with breastfeeding [88]. Despite no studies, the acceptance of teleworking following the COVID-19 pandemic could also help women to breastfeed [89]. Interestingly, the guarantee of paid breastfeeding breaks for at least six months has been shown to be associated with an increase of nearly 9% in exclusive breastfeeding [90]. Some workplace variables seem to be strongly associated with breastfeeding after RTW. Based on the literature, workplace support seems to be an important influence on breastfeeding duration after returning to work. Managerial and organizational support increases exclusive breastfeeding duration nearly twofold [91], with co-workers’ support being essential in the decision to continue breastfeeding [92]. Lack of breastfeeding facilities, such as a room dedicated for breastfeeding or a fridge, is associated with breastfeeding discontinuation after RTW [93]. Even if some laws promote breastfeeding at work, such as the Federal Break Time for Nursing Mothers law requiring employers covered by the Fair Labor Standards Act (FLSA) to provide basic accommodations for breastfeeding mothers at work in the USA, these laws are still not fully applied [16] and need to be expanded worldwide. There is very limited or even inexistent literature on the putative sociodemographic and clinical factors linked to breastfeeding after RTW. However, the literature is vast on factors known to affect breastfeeding initiation and duration. Mothers over 35 years old have higher chance of breastfeeding initiation [21] and continuation at six months [94]. Single parents or mothers without support from their partner have levels of lower initiation [95]. Smoking mothers are also less likely to initiate breastfeeding [96,97], as well as those who had a cesarean section [98] or those with lower education levels [22]. Cesarean section and low income are also two factors that decrease the duration of breastfeeding [98,99]. A recent study in Oceania also demonstrated that most of the previous factors are also risk factors for stopping full breastfeeding [100]. No data were found to indicate if infant sex influences breastfeeding initiation or duration. Multiparous women are more likely to breastfeed for six months or more [23], and by consequence are more likely to continue breastfeeding. Although controversial [101], among the other risk factors of non-breastfeeding are, research suggests, maternal obesity [94], not attending childbirth education [94], depression [94], or dyad connection [97].

## 5. Limitations

All meta-analyses have limitations [102]. Meta-analyses inherit the limitations of the individual studies of which they are composed and are subjected to a bias of selection of included studies. However, the use of broader keywords in the search strategy limited the number of missing studies. Despite our rigorous criteria for including studies in our meta-analysis, their quality varied. Most cross-sectional studies included in our meta-analyses described a bias of self-report, such as skipping questions and incomplete information, nondisclosure, and uncertainty regarding the timing of the questionnaire. Though there were similarities between the inclusion criteria, they were not identical. In particular, some studies included only mothers who worked the year before delivery, whereas other studies did not specify [47,49,50,54,55]. Two studies only included women who initiated breastfeeding [47,54], which may have led to a comparison bias; however, sensitivity analyses without these two studies did not influence the results. Moreover, our meta-analysis was based on a moderate number of studies, especially for exclusive breastfeeding. An important finding of our study is also the lack of breastfeeding data after RTW—some continents had no data available. Stratification by ethnicity was not feasible because of the lack of data; however, stratification by country/continent enabled international comparisons and should have taken into account the influence of baseline breastfeeding rates. Furthermore, the dates of RTW were too heterogeneous to stratify for; the lack of included studies also precluded stratification by time—both of which may have impacted the comparisons between continents and GDP.

## 6. Conclusions

Despite the scarcity of data, the prevalence of breastfeeding after returning to work is 25% and widely heterogeneous across the world. Even if economic status plays a role in breastfeeding after return to work, cultural aspects seem an important determinant, influencing public health policies and workplace breastfeeding support. We also showed the lack of data regarding breastfeeding after returning to work in most countries, with no data available from some continents such as Africa, demonstrating the strong need for data in these countries to inform effective preventive strategies.

## Figures and Tables

**Table 1:** Characteristics of included studies. * Adjusted by years of the study.

Study	Country	Type of Study	Follow-Up	Population	Recruitment Procedures	Occupation	GDP per Capita * (in $)	Type of Breastfeeding	Timing of Returning to Work	Other Parameters Measured
**Abou-ElWafa 2019** [44]	Egypt	Cross-sectional study	July–December 2017	633	All working mothers attending healthcare facilities	Professional/semi-professional; manual; business worker	2413	Exclusive	<4 months; 4 months	Maternal sociodemographics, employment patterns, and birth characteristics
**Aikawa 2015** [45]	Thailand	Cross-sectional study	February 2008	84	Mothers who visited the breastfeeding mobile clinic at a nursery goods exhibition in Bangkok	Government and semi-government; private company employee; self-employed or family business owner	4379	Non-exclusive	<3 months	Maternal sociodemographics, employment patterns, and birth characteristics
**Bergman 1981** [46]	Israel	Cross-sectional study	1979	291	Working women interviewed 7–9 months after delivery	All workers	5674	Non-exclusive	<3 months; 3 months; 4 months; 4–5 months; 5 months; 6 months	Maternal sociodemographics and employment patterns
**Bonet 2013** [47]	France	Cohort study	2003–2006	979	From EDEN mother–child cohort; pregnant women were recruited from the maternity wards of the Poitiers and Nancy University hospitals	All workers	34,760	Exclusive and non-exclusive	≤4 months; 5–8 months	Maternal sociodemographics and employment patterns
**Boralingiah 2016** [48]	India	Cross-sectional study	January–December 2014	107	Working mothers of the children attending the immunization center at JSS Hospital, Mysuru	All workers	1576	Exclusive	<6 months; >6 months	Maternal sociodemographics, employment patterns, and hospital breastfeeding practice
**Chuang 2010** [49]	Taiwan	Cohort study	2006–2007	20,172	From the Taiwan Birth Cohort Study	All workers	30,100	Non-exclusive	≤1 month; ≤2 months; ≤3 months; ≤6 months; ≤12 months	Maternal sociodemographics, employment patterns, birth characteristics, and hospital feeding practices
**Cox 2015** [50]	Australia	Cross-sectional study	2010–2011	427	Mothers recruited from maternity services in rural western Australia	All workers	51,937	Exclusive and non-exclusive	<6 months; 6–12 months	Maternal sociodemographics, employment patterns, birth characteristics, hospital feeding practices, and psychosocial factors
**Hawkins 2007** [51]	U.K.	Cohort study	September 2000–January 2002	6917	From the Millennium Cohort Study	Employed workers in the formal or informal sector	27,427	Non-exclusive	<3 months; 4 months	Maternal sociodemographics and employment patterns
**Jacknowitz 2008** [52]	USA	Cohort study	1989–1999	1506	From the National Longitudinal Survey of Youth and the Children of the National Longitudinal Survey	All workers	24,405	Non-exclusive	<6 weeks; >6 weeks and ≤3 months; >3 months and ≤6 months	Maternal sociodemographics, employment patterns, and birth characteristics
**Ogbuanu 2011** [53]	USA	Cohort study	2001–2003	6150	Data drawn from the Early Childhood Longitudinal Study–Birth Cohort	All workers	39,677	Exclusive and non-exclusive	<6 weeks; <3 months	Maternal sociodemographics, employment patterns, birth characteristics, and hospital feeding practices
**Piper 1996** [54]	USA	Cohort study	January 1989–June 1991	2372	Data from the 1988 National Maternal-Infant Health Survey	All workers	24,405	Exclusive and non-exclusive	<6 weeks; 6 weeks–3 months; after 3 months and up to 6 months	Maternal sociodemographics and employment patterns
**Scott 2006** [55]	Australia	Cohort study	September 2002–July 2003	587	Mothers contacted within the 3 days after birth from 2 maternity hospitals in Perth	All workers	23,437	Exclusive and non-exclusive	<6 months; 6–12 month	Maternal sociodemographics, employment patterns, birth characteristics, hospital feeding practices, and psychosocial factors
**Xiang 2016** [56]	Australia	Cross-sectional study	November 2010–February 2011	2300	Data from the BaselineMothers Survey	Paid employment	51,937	Exclusive and non-exclusive	<3 months; 3–6 months; <8 weeks; 9–16 weeks	Maternal sociodemographics and employment patterns
**Yimyam 1999** [57]	Thailand	Cross-sectional study	July–August 1994 and April–November 1995	295	Women approached in the growth monitoring clinic at Chiang Mai University Hospital or at Chiang Mai University’s Child Care Centre	Formal sector (public and private employee) and informal sector (pieceworker at home and self/family employed)	2845	Exclusive and non-exclusive	6 months	Maternal sociodemographics and employment patterns

## Data Availability

All relevant data were included in the paper.

## Appendix A

### Appendix A.1. Details for the Search Strategy Used within Each Database

**Pubmed**

“breast feeding” [MH] OR “feeding breast” [TW] OR “lactation” [TW] OR “breastfeeding” [TW] OR “breast feeding” [TW] OR “breast fed” [TW] OR breastfeed[TW] OR “breast feed” [TW] OR (breast[Title] AND feeding[Title]) OR “Breast Milk” [TW]

AND

“women, working” [MH] OR “work” [MH] OR “labor” [TW] OR “job” [TW] OR work[TW] OR works[TW] OR work’s[TW] OR Worker* [TW] OR working[TW] OR Worksite* [TW] OR workstation[TW] OR “workplace” [MH] OR workplace* [TW] OR occupation* [TW] OR employee* [TW] OR “employment” [MH] “employment” [TW] OR “return to work” [MH] OR “return to work” [TW] OR “return-to-work” [TW] OR “back to work” [TW] OR “back to works” [TW] OR (return[TW] OR returning[TW] OR back[TW]) AND (work[TW] OR job[TW] OR employ* [TW])

Filter Language = none

Filter Dates = none

*Results* = 960

**Cochrane Library**

“feeding breast”:ti,ab,kw OR “lactation”:ti,ab,kw OR “breastfeeding”:ti,ab,kw OR “breast feeding”:ti,ab,kw OR “breast fed”:ti,ab,kw OR breastfeed:ti,ab,kw OR “breast feed”:ti,ab,kw OR “Breast Milk”:ti,ab,kw OR (breast:ti AND feeding:ti)

AND

job:ti,ab,kw OR work:ti,ab,kw OR works:ti,ab,kw OR work’s:ti,ab,kw OR Worker*:ti,ab,kw OR working:ti,ab,kw OR Worksite*:ti,ab,kw OR workstation:ti,ab,kw OR workplace*:ti,ab,kw OR occupation*:ti,ab,kw OR employe*:ti,ab,kw OR “employment”:ti,ab,kw

Filter Language = not available in CENTRAL

Filter Dates = none

*Results* = 612

**Base**

(“breast feeding” lactation breastfeeding “breast fed” breastfeed “breast feed” “breast milk”)

AND

(Work works work’s Worker Workers working worksite worksites workstation workplace workplaces occupation occupational employee employees employed employment “return to job”)

Filter Language = none

Filter Dates = none

*Results* = 151

**Embase**

‘breast feeding’/exp AND ‘work’/exp AND [female]/lim

Filter Language = none

Filter Dates = none

*Results* = 109

### Appendix A.2. PRISMA Checklist

**Section/Topic**	**#**	**Checklist Item**	**Reported on Page #**
**TITLE**			
Title	1	Identify the report as a systematic review, meta-analysis, or both.	1
**ABSTRACT**			
Structured summary	2	Provide a structured summary including, as applicable: background; objectives; data sources; study eligibility criteria, participants, and interventions; study appraisal and synthesis methods; results; limitations; conclusions and implications of key findings; systematic review registration number.	2
**INTRODUCTION**			
Rationale	3	Describe the rationale for the review in the context of what is already known.	3
Objectives	4	Provide an explicit statement of questions being addressed with reference to participants, interventions, comparisons, outcomes, and study design (PICOS).	3
**METHODS**			
Protocol and registration	5	Indicate if a review protocol exists, if and where it can be accessed (e.g., Web address), and, if available, provide registration information including registration number.	3–6
Eligibility criteria	6	Specify study characteristics (e.g., PICOS, length of follow-up) and report characteristics (e.g., years considered, language, publication status) used as criteria for eligibility, giving rationale.	3–6
Information sources	7	Describe all information sources (e.g., databases with dates of coverage, contact with study authors to identify additional studies) in the search and date last searched.	3–6
Search	8	Present full electronic search strategy for at least one database, including any limits used, such that it could be repeated.	3–6 and Figure
Study selection	9	State the process for selecting studies (i.e., screening, eligibility, included in systematic review, and, if applicable, included in the meta-analysis).	3–6
Data collection process	10	Describe method of data extraction from reports (e.g., piloted forms, independently, in duplicate) and any processes for obtaining and confirming data from investigators.	3–6
Data items	11	List and define all variables for which data were sought (e.g., PICOS, funding sources) and any assumptions and simplifications made.	3–6
Risk of bias in individual studies	12	Describe methods used for assessing risk of bias of individual studies (including specification of whether this was done at the study or outcome level), and how this information is to be used in any data synthesis.	3–6
Summary measures	13	State the principal summary measures (e.g., risk ratio, difference in means).	3–6
Synthesis of results	14	Describe the methods of handling data and combining results of studies, if done, including measures of consistency (e.g., I^2^) for each meta-analysis.	3–6
Risk of bias across studies	15	Specify any assessment of risk of bias that may affect the cumulative evidence (e.g., publication bias, selective reporting within studies).	3–6
Additional analyses	16	Describe methods of additional analyses (e.g., sensitivity or subgroup analyses, meta-regression), if done, indicating which were pre-specified.	3–6
**RESULTS**			
Study selection	17	Give numbers of studies screened, assessed for eligibility, and included in the review, with reasons for exclusions at each stage, ideally with a flow diagram.	6–10
Study characteristics	18	For each study, present characteristics for which data were extracted (e.g., study size, PICOS, follow-up period) and provide the citations.	6–10 and Figures
Risk of bias within studies	19	Present data on risk of bias of each study and, if available, any outcome level assessment (see item 12).	6–10 and Figures
Results of individual studies	20	For all outcomes considered (benefits or harms), present, for each study: (a) simple summary data for each intervention group (b) effect estimates and confidence intervals, ideally with a forest plot.	6–10 and Figures
Synthesis of results	21	Present results of each meta-analysis done, including confidence intervals and measures of consistency.	6–10 and Figures
Risk of bias across studies	22	Present results of any assessment of risk of bias across studies (see Item 15).	6–10 and Figures
Additional analysis	23	Give results of additional analyses, if done (e.g., sensitivity or subgroup analyses, meta-regression [see Item 16]).	6–10
**DISCUSSION**			
Summary of evidence	24	Summarize the main findings including the strength of evidence for each main outcome; consider their relevance to key groups (e.g., healthcare providers, users, and policy makers).	10–13
Limitations	25	Discuss limitations at study and outcome level (e.g., risk of bias), and at review-level (e.g., incomplete retrieval of identified research, reporting bias).	14
Conclusions	26	Provide a general interpretation of the results in the context of other evidence, and implications for future research.	14
**FUNDING**			
Funding	27	Describe sources of funding for the systematic review and other support (e.g., supply of data); role of funders for the systematic review.	15–16

## References

[1] Horta B.L., de Mola C.L., Victora C.G. (2015). Breastfeeding and Intelligence: A Systematic Review and Meta-Analysis. Acta Paediatr. Oslo Nor. 1992.

[2] Chowdhury R., Sinha B., Sankar M.J., Taneja S., Bhandari N., Rollins N., Bahl R., Martines J. (2015). Breastfeeding and Maternal Health Outcomes: A Systematic Review and Meta-Analysis. Acta Paediatr. Oslo Nor. 1992.

[3] Victora C.G., Bahl R., Barros A.J.D., França G.V.A., Horton S., Krasevec J., Murch S., Sankar M.J., Walker N., Rollins N.C. (2016). Breastfeeding in the 21st Century: Epidemiology, Mechanisms, and Lifelong Effect. Lancet Lond. Engl..

[4] Turck D., Vidailhet M., Bocquet A., Bresson J.-L., Briend A., Chouraqui J.-P., Darmaun D., Dupont C., Frelut M.-L., Girardet J.-P. (2013). Breastfeeding: Health benefits for child and mother. Arch. Pediatr. Organe Off. Soc. Francaise Pediatr..

[5] Ball T.M., Bennett D.M. (2001). The Economic Impact of Breastfeeding. Pediatr. Clin. North Am..

[6] Kendall-Tackett K.A., Sugarman M. (1995). The Social Consequences of Long-Term Breastfeeding. J. Hum. Lact. Off. J. Int. Lact. Consult. Assoc..

[7] Krol K.M., Grossmann T. (2018). Psychological Effects of Breastfeeding on Children and Mothers. Bundesgesundh. Gesundh. Gesundh..

[8] World Health Organization (2005). The World Health Report. 2005: Make Every Mother and Child Count.

[9] Chatterji P., Frick K.D. (2005). Does Returning to Work After Childbirth AffectBreastfeeding Practices?. Rev. Econ. Househ..

[10] Fein S.B., Roe B. (1998). The Effect of Work Status on Initiation and Duration of Breast-Feeding. Am. J. Public Health.

[11] Ong G., Yap M., Li F.L., Choo T.B. (2005). Impact of Working Status on Breastfeeding in Singapore: Evidence from the National Breastfeeding Survey 2001. Eur. J. Public Health.

[12] Visness C.M., Kennedy K.I. (1997). Maternal Employment and Breast-Feeding: Findings from the 1988 National Maternal and Infant Health Survey. Am. J. Public Health.

[13] Lakati A., Binns C., Stevenson M. (2002). The Effect of Work Status on Exclusive Breastfeeding in Nairobi. Asia. Pac. J. Public Health.

[14] Wang S. (2019). The Role of Gender Role Attitudes and Immigrant Generation in Ethnic Minority Women’s Labor Force Participation in Britain. Sex Roles.

[15] Paul M. (1997). Breastfeeding Practices in Indonesia. Zhonghua Minguo Xiao Er Ke Yi Xue Hui Za Zhi J..

[16] Sriraman N.K., Kellams A. (2016). Breastfeeding: What Are the Barriers? Why Women Struggle to Achieve Their Goals. J. Womens Health 2002.

[17] Chen Y.C., Wu Y.-C., Chie W.-C. (2006). Effects of Work-Related Factors on the Breastfeeding Behavior of Working Mothers in a Taiwanese Semiconductor Manufacturer: A Cross-Sectional Survey. BMC Public Health.

[18] Lubold A.M. (2017). The Effect of Family Policies and Public Health Initiatives on Breastfeeding Initiation among 18 High-Income Countries: A Qualitative Comparative Analysis Research Design. Int. Breastfeed. J..

[19] Rollins N.C., Bhandari N., Hajeebhoy N., Horton S., Lutter C.K., Martines J.C., Piwoz E.G., Richter L.M., Victora C.G. (2016). Lancet Breastfeeding Series Group Why Invest, and What It Will Take to Improve Breastfeeding Practices?. Lancet Lond. Engl..

[20] Kimbro R.T. (2006). On-the-Job Moms: Work and Breastfeeding Initiation and Duration for a Sample of Low-Income Women. Matern. Child Health J..

[21] Bonet M., L’hélias L.F., Blondel B. (2008). Exclusive and mixed breastfeeding in a maternity unit in France, 2003. Arch. Pediatr. Organe Off. Soc. Fr. Pediatr..

[22] Acharya P., Khanal V. (2015). The Effect of Mother’s Educational Status on Early Initiation of Breastfeeding: Further Analysis of Three Consecutive Nepal Demographic and Health Surveys. BMC Public Health.

[23] Hackman N.M., Schaefer E.W., Beiler J.S., Rose C.M., Paul I.M. (2015). Breastfeeding Outcome Comparison by Parity. Breastfeed. Med..

[24] Kosmala-Anderson J., Wallace L.M. (2006). Breastfeeding Works: The Role of Employers in Supporting Women Who Wish to Breastfeed and Work in Four Organizations in England. J. Public Health Oxf. Engl..

[25] Taveras E.M., Capra A.M., Braveman P.A., Jensvold N.G., Escobar G.J., Lieu T.A. (2003). Clinician Support and Psychosocial Risk Factors Associated with Breastfeeding Discontinuation. Pediatrics.

[26] Weber D., Janson A., Nolan M., Wen L.M., Rissel C. (2011). Female Employees’ Perceptions of Organisational Support for Breastfeeding at Work: Findings from an Australian Health Service Workplace. Int. Breastfeed. J..

[27] Scott J.A., Binns C.W. (1999). Factors Associated with the Initiation and Duration of Breastfeeding: A Review of the Literature. Breastfeed. Rev. Prof. Publ. Nurs. Mothers Assoc. Aust..

[28] Smith J., McIntyre E., Craig L., Javanparast S., Strazdins L., Mortensen K. (2013). Workplace Support Breastfeeding and Health. Fam. Matters.

[29] Tsai S.-Y. (2013). Impact of a Breastfeeding-Friendly Workplace on an Employed Mother’s Intention to Continue Breastfeeding after Returning to Work. Breastfeed. Med. Off. J. Acad. Breastfeed. Med..

[30] Mirkovic K.R., Perrine C.G., Scanlon K.S., Grummer-Strawn L.M. (2014). Maternity Leave Duration and Full-Time/Part-Time Work Status Are Associated with US Mothers’ Ability to Meet Breastfeeding Intentions. J. Hum. Lact. Off. J. Int. Lact. Consult. Assoc..

[31] Moher D., Liberati A., Tetzlaff J., Altman D.G. (2009). The PRISMA Group Preferred Reporting Items for Systematic Reviews and Meta-Analyses: The PRISMA Statement. PLoS Med..

[32] The Newcastle–Ottawa Scale (NOS) for Assessing the Quality of Non-Randomized Studies in Meta-Analysis. http://www.ohri.ca/programs/clinical_epidemiology/oxford.asp.

[33] Modesti P.A., Reboldi G., Cappuccio F.P., Agyemang C., Remuzzi G., Rapi S., Perruolo E., Parati G. (2016). ESH Working Group on CV Risk in Low Resource Settings Panethnic Differences in Blood Pressure in Europe: A Systematic Review and Meta-Analysis. PLoS ONE.

[34] Ollier M., Chamoux A., Naughton G., Pereira B., Dutheil F. (2014). Chest CT Scan Screening for Lung Cancer in Asbestos Occupational Exposure: A Systematic Review and Meta-Analysis. Chest.

[35] Lanhers C., Pereira B., Naughton G., Trousselard M., Lesage F.-X., Dutheil F. (2015). Creatine Supplementation and Lower Limb Strength Performance: A Systematic Review and Meta-Analyses. Sports Med. Auckl. NZ.

[36] Lanhers C., Pereira B., Naughton G., Trousselard M., Lesage F.-X., Dutheil F. (2017). Creatine Supplementation and Upper Limb Strength Performance: A Systematic Review and Meta-Analysis. Sports Med. Auckl. NZ.

[37] Navel V., Mulliez A., Benoist d’Azy C., Baker J.S., Malecaze J., Chiambaretta F., Dutheil F. (2019). Efficacy of Treatments for Demodex Blepharitis: A Systematic Review and Meta-Analysis. Ocul. Surf..

[38] d’Azy C.B., Pereira B., Naughton G., Chiambaretta F., Dutheil F. (2016). Antibioprophylaxis in Prevention of Endophthalmitis in Intravitreal Injection: A Systematic Review and Meta-Analysis. PLoS ONE.

[39] Benichou T., Pereira B., Mermillod M., Tauveron I., Pfabigan D., Maqdasy S., Dutheil F. (2018). Heart Rate Variability in Type 2 Diabetes Mellitus: A Systematic Review and Meta-Analysis. PLoS ONE.

[40] Benoist d’Azy C., Pereira B., Chiambaretta F., Dutheil F. (2016). Oxidative and Anti-Oxidative Stress Markers in Chronic Glaucoma: A Systematic Review and Meta-Analysis. PLoS ONE.

[41] Courtin R., Pereira B., Naughton G., Chamoux A., Chiambaretta F., Lanhers C., Dutheil F. (2016). Prevalence of Dry Eye Disease in Visual Display Terminal Workers: A Systematic Review and Meta-Analysis. BMJ Open.

[42] DerSimonian R., Laird N. (1986). Meta-Analysis in Clinical Trials. Control. Clin. Trials.

[43] Russo M.W. (2007). How to Review a Meta-Analysis. Gastroenterol. Hepatol..

[44] Abou-ElWafa H.S., El-Gilany A.-H. (2019). Maternal Work and Exclusive Breastfeeding in Mansoura, Egypt. Fam. Pract..

[45] Aikawa T., Pavadhgul P., Chongsuwat R., Sawasdivorn S., Boonshuyar C. (2015). Maternal Return to Paid Work and Breastfeeding Practices in Bangkok, Thailand. Asia. Pac. J. Public Health.

[46] Bergman R., Feinberg D. (1981). Working Women and Breastfeeding in Israel. J. Adv. Nurs..

[47] Bonet M., Marchand L., Kaminski M., Fohran A., Betoko A., Charles M.-A., Blondel B. (2013). “EDEN Mother–Child Cohort Study Group” Breastfeeding Duration, Social and Occupational Characteristics of Mothers in the French “EDEN Mother-Child” Cohort. Matern. Child Health J..

[48] Boralingiah P., Polineni V., Kulkarni P., Manjunath R. (2016). Study of Breastfeeding Practices among Working Women Attending a Tertiary Care Hospital, Mysore, Karnataka, India. Int. J. Community Med. Public Health.

[49] Chuang C.-H., Chang P.-J., Chen Y.-C., Hsieh W.-S., Hurng B.-S., Lin S.-J., Chen P.-C. (2010). Maternal Return to Work and Breastfeeding: A Population-Based Cohort Study. Int. J. Nurs. Stud..

[50] Cox K., Binns C.W., Giglia R. (2015). Predictors of Breastfeeding Duration for Rural Women in a High-Income Country: Evidence from a Cohort Study. Acta Paediatr. Oslo Nor. 1992.

[51] Hawkins S.S., Griffiths L.J., Dezateux C., Law C. (2007). Millennium Cohort Study Child Health Group The Impact of Maternal Employment on Breast-Feeding Duration in the UK Millennium Cohort Study. Public Health Nutr..

[52] Jacknowitz A. (2008). The Role of Workplace Characteristics in Breastfeeding Practices. Women Health.

[53] Ogbuanu C., Glover S., Probst J., Liu J., Hussey J. (2011). The Effect of Maternity Leave Length and Time of Return to Work on Breastfeeding. Pediatrics.

[54] Piper S., Parks P.L. (1996). Predicting the Duration of Lactation: Evidence from a National Survey. Birth.

[55] Scott J.A., Binns C.W., Oddy W.H., Graham K.I. (2006). Predictors of Breastfeeding Duration: Evidence from a Cohort Study. Pediatrics.

[56] Xiang N., Zadoroznyj M., Tomaszewski W., Martin B. (2016). Timing of Return to Work and Breastfeeding in Australia. Pediatrics.

[57] Yimyam S., Morrow M., Srisuphan W. (1999). Role Conflict and Rapid Socio-Economic Change: Breastfeeding among Employed Women in Thailand. Soc. Sci. Med. 1982.

[58] World Development Indicators | DataBank. https://databank.worldbank.org/source/world-development-indicators.

[59] WHO Technical Staff Continued Breastfeeding for Healthy Growth and Development of Children. http://www.who.int/elena/titles/bbc/continued_breastfeeding/en/.

[60] Brown C.R.L., Dodds L., Legge A., Bryanton J., Semenic S. (2014). Factors Influencing the Reasons Why Mothers Stop Breastfeeding. Can. J. Public Health Rev. Can. Sante Publique.

[61] Thomas-Jackson S.C., Bentley G.E., Keyton K., Reifman A., Boylan M., Hart S.L. (2016). In-Hospital Breastfeeding and Intention to Return to Work Influence Mothers’ Breastfeeding Intentions. J. Hum. Lact. Off. J. Int. Lact. Consult. Assoc..

[62] Ibanez G., Martin N., Denantes M., Saurel-Cubizolles M.-J., Ringa V., Magnier A.-M. (2012). Prevalence of Breastfeeding in Industrialized Countries. Rev. Epidemiol. Sante Publique.

[63] Walters D., Horton S., Siregar A.Y.M., Pitriyan P., Hajeebhoy N., Mathisen R., Phan L.T.H., Rudert C. (2016). The Cost of Not Breastfeeding in Southeast Asia. Health Policy Plan..

[64] COAG Health Council Australian National Breastfeeding Strategy 2019 and Beyond. http://www.coaghealthcouncil.gov.au/Publications/Reports.

[65] Alzaheb R.A. (2017). A Review of the Factors Associated with the Timely Initiation of Breastfeeding and Exclusive Breastfeeding in the Middle East. Clin. Med. Insights Pediatr..

[66] Wagner S., Kersuzan C., Gojard S., Tichit C., Nicklaus S., Thierry X., Charles M.A., Lioret S., de Lauzon-Guillain B. (2019). Breastfeeding Initiation and Duration in France: The Importance of Intergenerational and Previous Maternal Breastfeeding Experiences—Results from the Nationwide ELFE Study. Midwifery.

[67] Negin J., Coffman J., Vizintin P., Raynes-Greenow C. (2016). The Influence of Grandmothers on Breastfeeding Rates: A Systematic Review. BMC Pregnancy Childbirth.

[68] Knight C.R., Brinton M.C. (2017). One Egalitarianism or Several? Two Decades of Gender-Role Attitude Change in Europe. Am. J. Sociol..

[69] McIntyre E., Pisaniello D., Gun R., Sanders C., Frith D. (2002). Balancing Breastfeeding and Paid Employment: A Project Targeting Employers, Women and Workplaces. Health Promot. Int..

[70] Sydney Local Health District Breastfeeding Guidelines. https://www.cesphn.org.au/images/SLHD_BF_guidelines_2014.pdf.

[71] South Western Sydney Local Health District a Research Strategy for South Western Sydney Local Health District. https://www.swslhd.health.nsw.gov.au/planning/content/pdf/researchissuespaper.pdf.

[72] South Western Sydney Local Health District the Maternal Early Childhood Sustained Home-Visiting (MECSH) Program. https://www.earlychildhoodconnect.edu.au/home-visiting-programs/mecsh-public/about-mecsh.

[73] Guendelman S., Kosa J.L., Pearl M., Graham S., Goodman J., Kharrazi M. (2009). Juggling Work and Breastfeeding: Effects of Maternity Leave and Occupational Characteristics. Pediatrics.

[74] Ryan A.S., Zhou W., Arensberg M.B. (2006). The Effect of Employment Status on Breastfeeding in the United States. Womens Health Issues Off. Publ. Jacobs Inst. Womens Health.

[75] Navarro-Rosenblatt D., Garmendia M.-L. (2018). Maternity Leave and Its Impact on Breastfeeding: A Review of the Literature. Breastfeed. Med. Off. J. Acad. Breastfeed. Med..

[76] Mandal B., Roe B.E., Fein S.B. (2010). The Differential Effects of Full-Time and Part-Time Work Status on Breastfeeding. Health Policy Amst. Neth..

[77] Ayton J.E., Tesch L., Hansen E. (2019). Women’s Experiences of Ceasing to Breastfeed: Australian Qualitative Study. BMJ Open.

[78] Hangchaovanich Y., Voramongkol N. (2006). Breastfeeding Promotion in Thailand. J. Med. Assoc. Thail. Chotmaihet Thangphaet.

[79] Hmone M.P., Li M., Alam A., Dibley M.J. (2017). Mobile Phone Short Messages to Improve Exclusive Breastfeeding and Reduce Adverse Infant Feeding Practices: Protocol for a Randomized Controlled Trial in Yangon, Myanmar. JMIR Res. Protoc..

[80] Health and Human Services Letter. https://media.washingtonpost.com/wp-srv/health/documents/yeutterletters.pdf.

[81] Murtagh L., Moulton A.D. (2011). Working Mothers, Breastfeeding, and the Law. Am. J. Public Health.

[82] USBC: Healthy People 2020: Breastfeeding Objectives. http://www.usbreastfeeding.org/p/cm/ld/fid=221.

[83] Garvin C.C., Sriraman N.K., Paulson A., Wallace E., Martin C.E., Marshall L. (2013). The Business Case for Breastfeeding: A Successful Regional Implementation, Evaluation, and Follow-Up. Breastfeed. Med. Off. J. Acad. Breastfeed. Med..

[84] Noble S. (2001). Maternal Employment and the Initiation of Breastfeeding. Acta Paediatr..

[85] Skafida V. (2012). Juggling Work and Motherhood: The Impact of Employment and Maternity Leave on Breastfeeding Duration: A Survival Analysis on Growing Up in Scotland Data. Matern. Child Health J..

[86] Nardi A.L., von Frankenberg A.D., Franzosi O.S., do Espirito Santo L.C. (2020). Impact of institutional aspects on breastfeeding for working women: A systematic review. Cienc. Saude Coletiva.

[87] Johnston M.L., Esposito N. (2007). Barriers and Facilitators for Breastfeeding among Working Women in the United States. J. Obstet. Gynecol. Neonatal Nurs. Jognn.

[88] Vilar-Compte M., Hernández-Cordero S., Ancira-Moreno M., Burrola-Méndez S., Ferre-Eguiluz I., Omaña I., Pérez Navarro C. (2021). Breastfeeding at the Workplace: A Systematic Review of Interventions to Improve Workplace Environments to Facilitate Breastfeeding among Working Women. Int. J. Equity Health.

[89] Morilla-Luchena A., Muñoz-Moreno R., Chaves-Montero A., Vázquez-Aguado O. (2021). Telework and Social Services in Spain during the COVID-19 Pandemic. Int. J. Environ. Res. Public. Health.

[90] Heymann J., Raub A., Earle A. (2013). Breastfeeding Policy: A Globally Comparative Analysis. Bull. World Health Organ..

[91] Scott V.C., Taylor Y.J., Basquin C., Venkitsubramanian K. (2019). Impact of Key Workplace Breastfeeding Support Characteristics on Job Satisfaction, Breastfeeding Duration, and Exclusive Breastfeeding Among Health Care Employees. Breastfeed. Med. Off. J. Acad. Breastfeed. Med..

[92] Zhuang J., Bresnahan M.J., Yan X., Zhu Y., Goldbort J., Bogdan-Lovis E. (2019). Keep Doing the Good Work: Impact of Coworker and Community Support on Continuation of Breastfeeding. Health Commun..

[93] Amin R.M., Said Z.M., Sutan R., Shah S.A., Darus A., Shamsuddin K. (2011). Work Related Determinants of Breastfeeding Discontinuation among Employed Mothers in Malaysia. Int. Breastfeed. J..

[94] Forster D.A., McLachlan H.L., Lumley J. (2006). Factors Associated with Breastfeeding at Six Months Postpartum in a Group of Australian Women. Int. Breastfeed. J..

[95] Sullivan M.L., Leathers S.J., Kelley M.A. (2004). Family Characteristics Associated with Duration of Breastfeeding during Early Infancy among Primiparas. J. Hum. Lact. Off. J. Int. Lact. Consult. Assoc..

[96] Ludvigsson J.F., Ludvigsson J. (2005). Socio-Economic Determinants, Maternal Smoking and Coffee Consumption, and Exclusive Breastfeeding in 10,205 Children. Acta Paediatr. Oslo Nor. 1992.

[97] Cohen S.S., Alexander D.D., Krebs N.F., Young B.E., Cabana M.D., Erdmann P., Hays N.P., Bezold C.P., Levin-Sparenberg E., Turini M. (2018). Factors Associated with Breastfeeding Initiation and Continuation: A Meta-Analysis. J. Pediatr..

[98] Hobbs A.J., Mannion C.A., McDonald S.W., Brockway M., Tough S.C. (2016). The Impact of Caesarean Section on Breastfeeding Initiation, Duration and Difficulties in the First Four Months Postpartum. BMC Pregnancy Childbirth.

[99] Li R., Darling N., Maurice E., Barker L., Grummer-Strawn L.M. (2005). Breastfeeding Rates in the United States by Characteristics of the Child, Mother, or Family: The 2002 National Immunization Survey. Pediatrics.

[100] Chimoriya R., Scott J.A., John J.R., Bhole S., Hayen A., Kolt G.S., Arora A. (2020). Determinants of Full Breastfeeding at 6 Months and Any Breastfeeding at 12 and 24 Months among Women in Sydney: Findings from the HSHK Birth Cohort Study. Int. J. Environ. Res. Public. Health.

[101] Mangrio E., Persson K., Bramhagen A.-C. (2018). Sociodemographic, Physical, Mental and Social Factors in the Cessation of Breastfeeding before 6 Months: A Systematic Review. Scand. J. Caring Sci..

[102] LeLorier J., Grégoire G., Benhaddad A., Lapierre J., Derderian F. (1997). Discrepancies between Meta-Analyses and Subsequent Large Randomized, Controlled Trials. N. Engl. J. Med..

